# Fine-Grained Distribution of a Non-Native Resource Can Alter the Population Dynamics of a Native Consumer

**DOI:** 10.1371/journal.pone.0143052

**Published:** 2015-11-17

**Authors:** Mifuyu Nakajima, Carol L. Boggs

**Affiliations:** 1 Department of Biology, Stanford University, Stanford, California, United States of America; 2 Rocky Mountain Biological Laboratory, Crested Butte, Colorado, United States of America; Indian Institute of Science, INDIA

## Abstract

New interactions with non-native species can alter selection pressures on native species. Here, we examined the effect of the spatial distribution of a non-native species, a factor that determines ecological and evolutionary outcomes but that is poorly understood, particularly on a fine scale. Specifically, we explored a native butterfly population and a non-native plant on which the butterfly oviposits despite the plant’s toxicity to larvae. We developed an individual-based model to describe movement and oviposition behaviors of each butterfly, which were determined by plant distribution and the butterfly's host preference genotype. We estimated the parameter values of the model from rich field data. We simulated various patterns of plant distributions and compared the rates of butterfly population growth and changes in the allele frequency of oviposition preference. Neither the number nor mean area of patches of non-native species affected the butterfly population, whereas plant abundance, patch shape, and distance to the nearest native and non-native patches altered both the population dynamics and genetics. Furthermore, we found a dramatic decrease in population growth rates when we reduced the distance to the nearest native patch from 147 m to 136 m. Thus changes in the non-native resource distribution that are critical to the fate of the native herbivore could only be detected at a fine-grained scale that matched the scale of a female butterfly’s movement. In addition, we found that the native butterfly population was unlikely to be rescued by the exclusion of the allele for acceptance of the non-native plant as a host. This study thus highlights the importance of including both ecological and evolutionary dynamics in analyses of the outcome of species interactions and provides insights into habitat management for non-native species.

## Introduction

Biological invasions by non-native species are increasing world-wide as a result of the increasing pace of global human movement [1, 2]. Many non-native species have radically changed the structure of invaded communities (e.g., [3]). Several factors determine the ecological and evolutionary outcome of interactions in invaded communities, including the local movement patterns of native and invading species, the scale of spatial distributions relative to those movement patterns, the spatial pattern of patch distribution, the extent to which the non-native species is detrimental or beneficial to the native species, and the genetics underlying the relationship between the two species. The effects of the spatial scale of movement relative to that of distributions are important for understanding the role of individual movements in determining population dynamics [4–6]. The effects of resource heterogeneity at coarser-grained scales, e.g. the effects of habitat quality, size, and connectivity on animal migration, metapopulation dynamics and gene flow, have been studied for decades. Likewise, analyses of animal movements on finer-grained scales, such as foraging, mating and patrolling of territories, have provided important ecological information including insights into habitat use, sociality, and spatial dynamics of populations [7, 8]. Here, we combined these two approaches by examining population and genetic dynamics with changing resource heterogeneity at the scale of local, small animal movements.

We used a system comprising a native butterfly population and a non-native plant on which the butterfly oviposits despite the fact that its larvae cannot develop on the plant. Patches of the non-native plant thus are sink habitats for butterflies and can negatively impact butterfly fitness [9]. Here, we modified plant abundance and distribution in a computer simulation in order to investigate how the native butterflies react to various spatial patterns of the invasive plants on a fine-grained scale. Specifically, we used an individual-based model (IBM) to describe the movement and oviposition behavior of individual butterflies, which was determined by plant distribution. Each individual butterfly was characterized by various life history parameters and a genotype for host preference. Movement and oviposition were also influenced by the phenology of both the plant and the butterfly: the butterflies disregarded a host plant with senescent leaves, and hosts with fresh leaves were only used during the butterfly’s flight season. Detailed data on butterfly life histories and movements as well as distributions of both plants and the butterfly are available to parameterize the model (described in Material and Methods).

To examine how the spatial distribution of the non-native host plant affects the butterfly population, we focused on several spatial attributes of plant distribution: plant abundance, the shape, size and the number of patches and distance to the other patches. We intuitively expect that the greater the density of the non-native plant, the greater the proportion of eggs laid on it, and the greater the decrease in the butterfly’s population size. However, the increased number of eggs on the detrimental host could also facilitate exclusion of the gene allowing acceptance of the non-native host, and promote population size increase. Our IBM enabled us to explore the dynamics of the relationship between the population growth rate and the change in frequency for the allele for acceptance of the detrimental, non-native host plant. Previous studies with coarser-grained scales suggested that patch shapes, sizes and distances between patches all affect dispersal and search for a new patch [6, 10, 11]. We tested the effects of those spatial attributes in a case where the sink and source patches were well mixed at a finer-grained scale.

## Materials and Methods

### Study system

**Fig 1:**
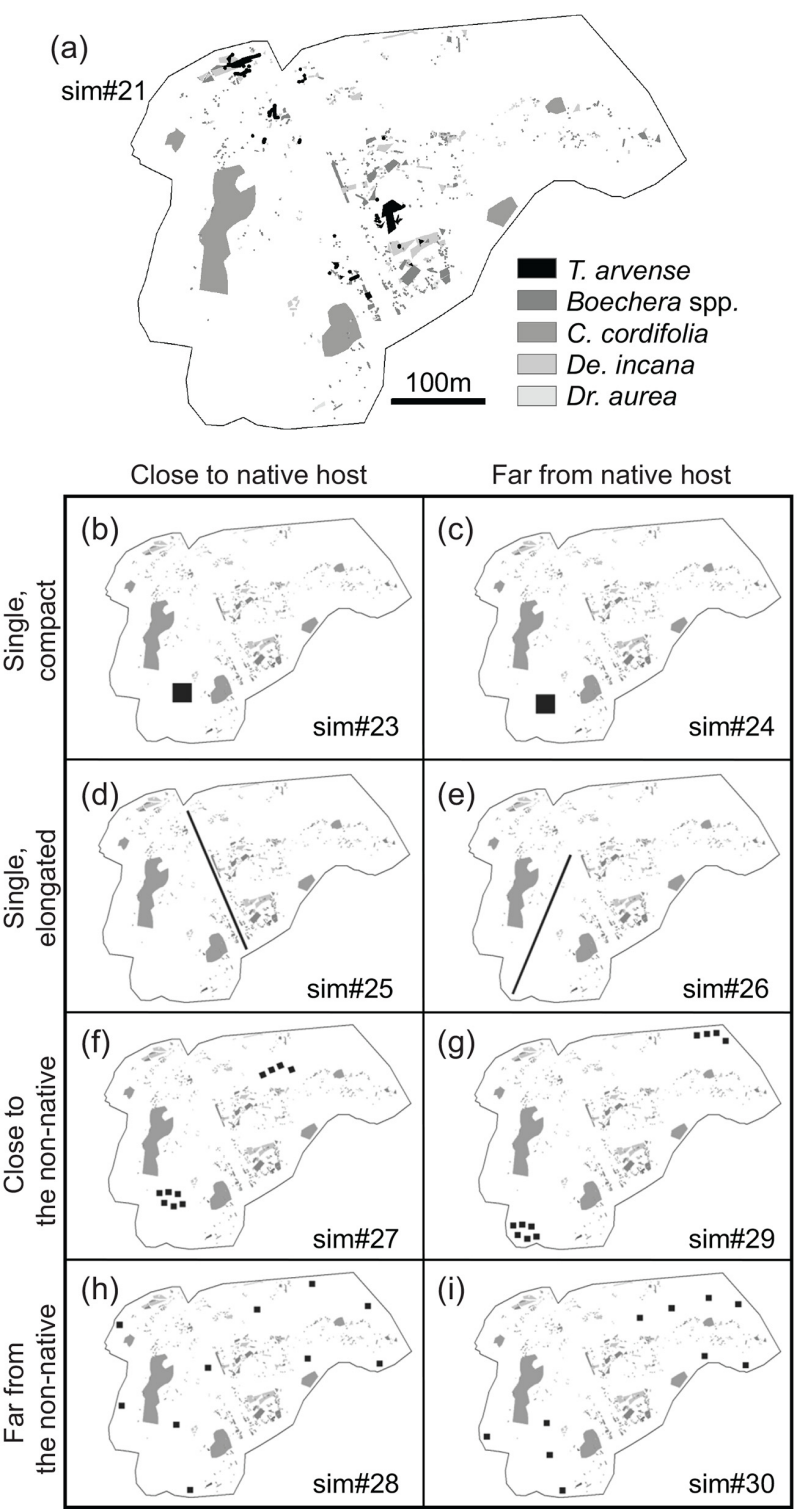
Simulated plant distribution patterns. The simulation number in each panel corresponds to the number in Fig 3. The patches of native plants were not modified from the observed data collected in 2000. The total patch area of the non-native host plant, *T*. *arvense*, in each panel was almost the same and was increased to approximately 400% of the patch area observed in 2000. The center of the patches of *T*. *arvense* in panel (a) were not moved from those observed in 2000. See the text for details of other distribution modifications.

Our study site was located at the Rocky Mountain Biological Laboratory (RMBL) in Gothic, Gunnison County, Colorado, USA (38° 57' 22.89” N, 106° 59' 18.23” W; elevation 2885 m above sea level, 16.1 ha). The study system consisted of a native butterfly, *Pieris macdunnoughii* (Pieridae; formerly *P*. *napi macdunnoughii*; see [12]), and its larval host plants, which include a non-native crucifer (Brassicaceae), *Thlaspi arvense* L., and four other native crucifers, *Boechera* spp. (formerly *Arabis* spp.), *Cardamine cordifolia* A. Gray, *Draba aurea* Vahl ex Hornem., and *Descurainia incana* (Bernh. ex Fisch. & C.A. Mey.) Dorn. Unlike *T*. *arvense*, the four native crucifers support *P*. *macdunnoughii*'s larval development [9, 13]. *Thlaspi arvense* is a Eurasian weed that arrived at RMBL in the mid to late 1800s [14]. It invades disturbed areas, which frequently occur within RMBL. Hereafter, “host plants” refers to the four native plant species plus *T*. *arvense*. The locations of all host plants that were visible were recorded in mid-July 2000 (Fig 1A, [9]). We refer to this plant distribution as the “observed” distribution. Habitat aridity, which was correlated with butterfly occupancy, was also estimated in 2000 (Fig 1A, [9]).

### Simulation flow

The IBM describes the movements of female *P*. *macdunnoughii* during their flight season, which occurs from approximately June until mid-August (54 days; Figure A in S1 Appendix). The host plant distribution at RMBL was represented by a series of fine grids (0.25 × 0.25 m cell), each containing the host plant (or none) that was the dominant species in the focal cell in the field survey (Fig 1A; [9]). Aridity levels (wet, intermediate, or dry) were also assigned to each cell (see [9] for the details). Based on the observed phenology, some plants senesced during the female butterfly flight season. All hosts were available in the first trimester of the flight season, *Boechera* spp. and *De*. *incana* senesced in the second trimester, and only *C*. *cordifolia* was available in the last trimester.

**Fig 2:**
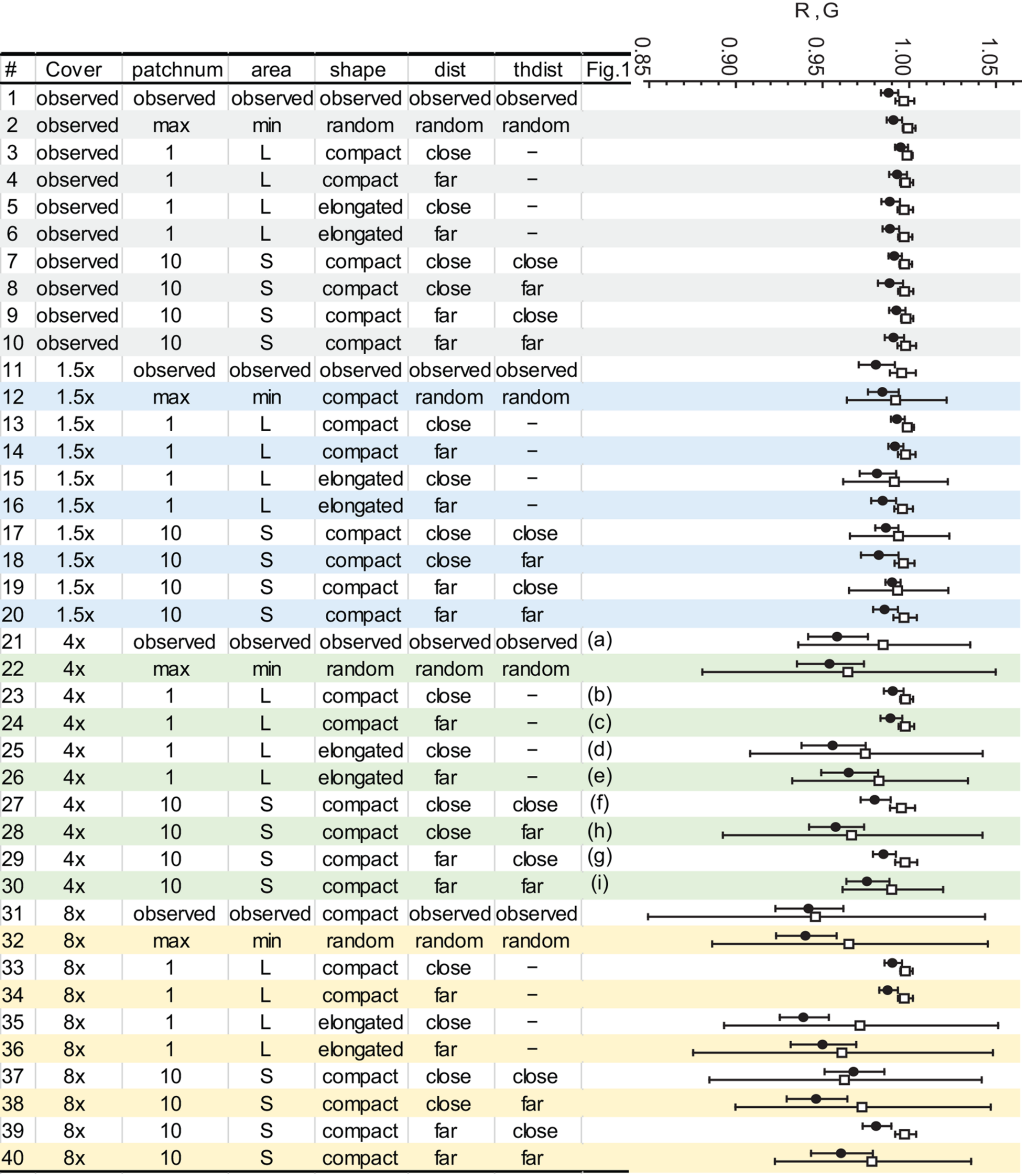
Simulation flow chart for the IBM.

The simulation flow is summarized in Fig 2, and the parameter values are in Table 1. Each individual butterfly was characterized by the genotype of oviposition preference (more details about the genotype are given below). Each female also had a given lifespan, which was the number of days since adult emergence, obtained randomly from an estimated probability distribution (Table 1; also see S1 Appendix for details of lifespan estimation). The lifespan included both death and emigration from the habitat. A female was excluded from the simulation at the end of her given lifespan. At the beginning of a flight season, we recorded the total number of emerging female butterflies in that year that survived from eggs laid by the previous generation. We then randomly selected emerging females for each day throughout the flying season based on the number of emerging females on each day determined by a β-function (Table 1, also see S1 Appendix for details of the emergence estimation). Females emerged on the scheduled day, and remaining females stayed inactive. An emerged female mated immediately with a male randomly chosen from the surviving eggs laid by the previous generation. In the first generation, adult females started in random cells occupied by native hosts. Adult females in subsequent generations started in the cell where they were oviposited and developed as larvae, because larvae have low mobility [13]. A mated female moves through a patchy habitat and oviposits if she stops at a cell that contains a suitable host. While searching for a suitable host, the distance that a female flies depends on whether she is currently on a host plant or not and on the aridity of the current location (Table 1; also see S1 Appendix for the details of estimation). The turning angle was random [15]. Host suitability depends on whether the host plant is native or not, whether the plant is senescent or not and the female’s genotype and motivation to oviposit. Oviposition choice is sex-linked (Boggs et al., unpublished manuscript), which in Lepidoptera means that it is determined by the paternal chromosome since females are heterogametic. Given that the details of the genetic system of oviposition preference are unknown, we assumed a 1-locus-2-allele system for simplicity. In the simulation, females oviposited on *T*. *arvense* in addition to the native hosts only if they had the allele to accept the plant. The genotype was determined by the genotypes of the fathers. We assumed that the adult genotypic distribution was at Hardy-Weinberg equilibrium. For example, if the father was a dominant homozygote Z_reject_Z_reject_ (where Z_reject_ and Z_accept_ represent whether the allele codes for rejecting or accepting *T*. *arvense*, respectively), the offspring's genotype was Z_reject_W if female and Z_reject_Z_reject_ or Z_reject_Z_accept_ if male, depending on the mother's genotype. The sex of the oviposited egg was determined randomly with a probability of 0.5. Females had a limited number of available eggs to lay each day and over a lifespan (Table 1, and see S1 Appendix for the details of estimation of daily egg load). The daily egg load depended on the female’s age. Females oviposited a single egg at a time [13]. In the simulation, a female on a suitable host oviposited there with a probability of 0.75. We set no limits for the number of eggs in a cell. A female butterfly stopped moving on a given day when either the total distance of her flight on that day reached the daily flight distance, which was chosen randomly from an estimated probability (Table 1; also see S1 Appendix for details), or she laid all the eggs for the day. Thus some eggs could be retained in the female and not oviposited. We assumed this had no effects on the subsequent egg load. When all the females met either of the conditions for stopping, the day was ended. At the start of the next day, females that reached their given lifespan were excluded from the simulation. Remaining females started moving from the cell where they stopped the previous day.

**Table 1:** Parameters and values of the model.

Parameter		Value
Adult lifespan		–(ln*x*)^–1^, with *x*~N(0.86, 0.04)
Survival from egg to adulthood		0.00651 on native host, 0 on *T*. *arvense*
Immigration rate		0.07
Initial allele frequency		0.5
Step size	(wet)	8 cells
	(intermediate)	10 cells
	(dry)	15 cells
	(oviposition)	6 cells
Daily flight distance		N(4134.54, 6.43×10^7^)
Min/max. flight distance		16.38/28800 m
Oviposition probability		0.75
Daily egg load		483.7 × lnN(1.69, 0.66)
Daily emergence probability		β-distribution with α = 1.75, β = 8.28

We assumed that the *P*. *macdunnoughii* population has one generation per year [15]. At the end of the flight season, we counted the eggs laid, and from those eggs we randomly selected the offspring that would survive until adult emergence, with the total number given by a probability including egg hatchability, larval survival, and pupation and adult emergence success (Table 1 and S1 Appendix). Larval survival is host plant-dependent; i.e., none survive on *T*. *arvense* [9, 13], while those on the native hosts survive with a given probability (see S1 Appendix for details of estimation). We introduced female immigrants each year. Immigrants were not defined as those who entered the habitat as adults, but rather those who emerged from eggs oviposited by females who entered the habitat in the previous generation. The number of immigrants was proportional to the focal population size, and the immigration rate was set at 7%. Immigrants started from randomly chosen cells occupied by native host plants. As mentioned above, emigration was combined with death and its probability was included in lifespan. There are no data separating death and emigration to our knowledge.

We started all the simulations with 150 females. We also explored how the intial population size affected the simulation results (see “Sensitivity analysis”). We ran each simulation 50 times for 100 generations each to obtain replicates. At the end of each simulation, we calculated the population growth rate *R*, which is the geometric mean of *N*_*t*_ / *N*_t−1_ (*N*_*t*_ denotes the population size at generation *t*), and the rate of allele frequency change *G*, which is the geometric mean of *p*_*t*_ / *p*_t−1_ (*p*_*t*_ denotes the frequency of allele Z_accept_ at generation *t*). Because the Z allele is on the sex chromosome, *p*_*t*_ in females is calculated as the frequency of genotype Z_accept_W, and *p*_*t*_ in males is [the frequency of genotype Z_accept_Z_accept_ + the frequency of genotype Z_reject_Z_accept_ /2]. The frequency *p*_*t*_ in the whole population is (2 *p*_*t*,*male*_ + *p*_*t*,*female*_) /3. All simulations were done using Mathematica 9.0 (Wolfram Research, Inc., IL, USA). The maps of plant distribution used in the simulation are provided online (10.6084/m9.figshare.1592706).

### Spatial pattern of *T*. *arvense*

**Fig 3:**
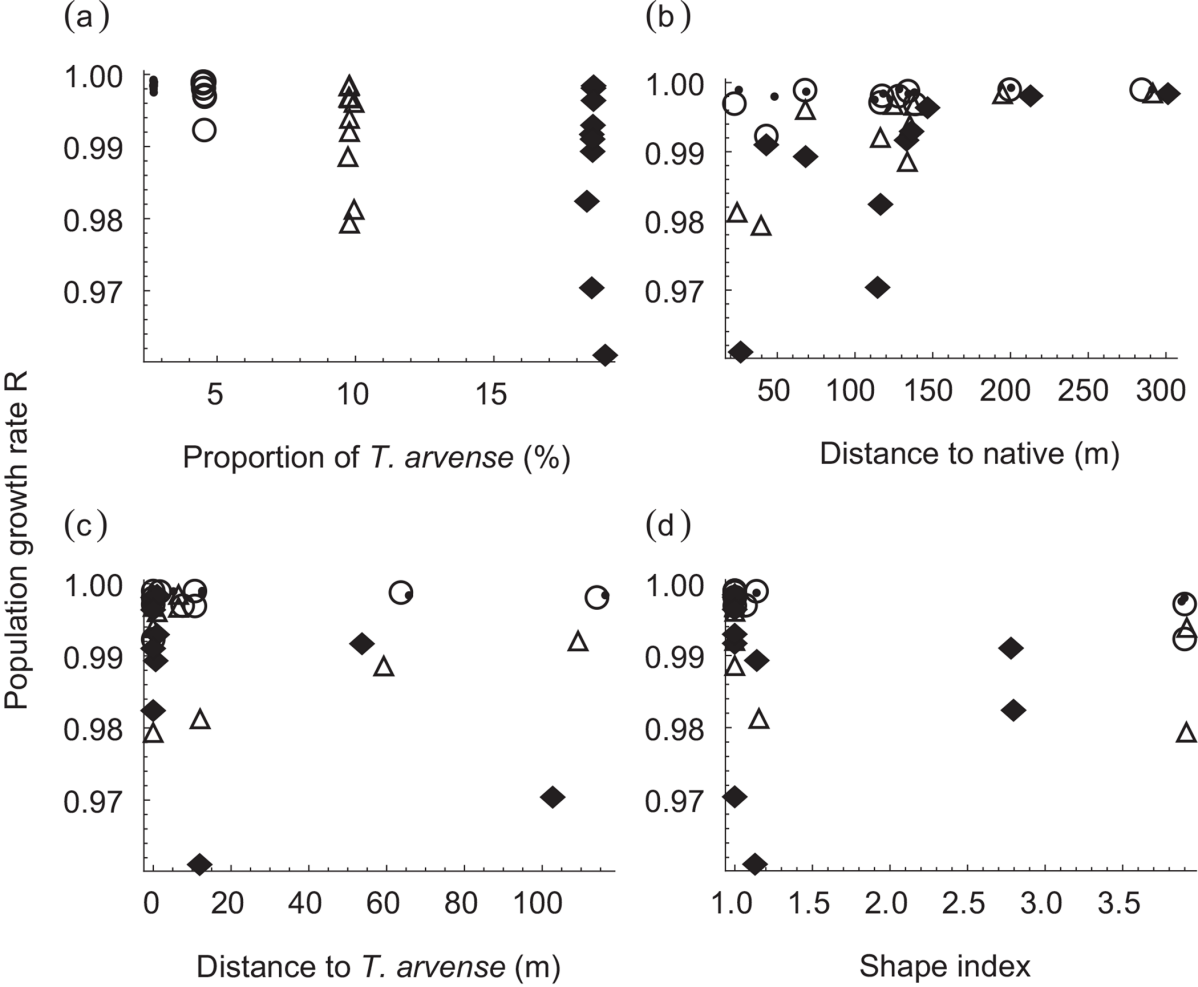
Spatial patterns of *Thlaspi arvense* simulated by the IBM (left) and the population growth rate *R* and the rate of allele frequency change *G* of each simulation, shown in closed circle and open square, respectively (right). The right-end column of the table shows the corresponding panel in Fig 1.

We changed the abundance and spatial distribution of *T*. *arvense* to test how the following spatial attributes of *T*. *arvense* affect the population and allele frequency dynamics of female butterflies: (1) total number of cells occupied by *T*. *arvense* (“*cover*”), (2) the number of patches (“*patchnum*”), (3) mean area of each patch (“*area*”), (4) patch compactness (“*shape*”), (5) distance to the nearest native host patch (“*dist*”), and (6) distance to the nearest *T*. *arvense* patch (“*thdist*”). These six attributes were changed as described below and simulations were run with all possible combinations, resulting in 40 simulation scenarios (Fig 3; also see Fig 1). The patches of native hosts were not changed throughout the simulations for simplicity.

#### (1) “*Cover*”

We enlarged each patch of *T*. *arvense* in the habitat without changing the central location of each patch to make the total proportion of *T*. *arvense* cover to total host cover as approximately 1.5, 4, and 8 times larger than the observed, which was 2.7%. When the enlarged patches of *T*. *arvense* conflicted with native plants, the cells in conflict were occupied by *T*. *arvense*. Note that when we enlarged the observed patches to increase the *T*. *arvense* cover, some of the close patches merged. These merged patches were counted as one patch, thus resulting in fewer patches in these simulations (i.e., 45, 26, and 22 patches when *cover* was 1.5-, 4- and 8-fold larger than the observed, respectively).

#### (2) Number of patches (“*patchnum*”) and (3) mean area of each patch (“*area*”)

We examined whether a single large or several small sink patches had a negative impact on the butterfly population. We constructed one and 10 square-shaped patches without changing the total *cover* (e.g., Fig 1B VS Fig 1F). The observed distribution had 59 *T*. *arvense* patches. As an extreme case of many patches, we assigned each *T*. *arvense* individual to a patch consisting of one cell, and randomly allocated those patches in vacant cells throughout the habitat. These distribution patterns are described as simulation number 2, 12, 22 and 32 in Fig 3, respectively. The number of patches (“*patchnum*”) in the Table were categorized as “max” and the “*area*” as “min” in Fig 3, because the cells occupied by *T*. *arvense* were randomly allocated one by one, resulting in the maximum number of patches possible and the minimum size of patches possible. The actual numbers and sizes of patches are shown in S1 Table. We regarded adjacent cells occupied by *T*. *arvense* as one patch.

#### (4) Patch compactness (“*shape*”)

We changed the shape of *T*. *arvense* patches to compact (e.g., Fig 1B) or elongated (e.g., Fig 1H). The shape algorithm, which measures compactness, was as follows: *Shape* = (0.25 [patch perimeter,m]) / √ [patch area, m^2^] [16].

#### (5) distance to the nearest native host patch (“*dist*”) and (6) to the nearest *T*. *arvense* patch (“*thdist*”)

We located the *T*. *arvense* close to and far from the native patches (e.g., Fig 1B VS 1C) and to each other (e.g., Fig 1F VS 1H). “Close” and “far” meant that the butterfly patches were closer to or farther, respectively, from the nearest patch on average than in the observed distribution. The distance to the nearest patch (m) shown in S1 Table was measured as the least distance between patch peripheries.

To maintain the same mean distance between the native and the non-native host patches and also the same mean distance among *T*. *arvense* patches within the “close” and “far” simulations, the simulated values were carefully selected. For example, a large single patch of *T*. *arvense* in a “far” from the native hosts shown in Fig 1C could be located closer to the periphery of the habitat to have a higher contrast to the simulation in Fig 1B. Instead, however, it was located at approximately the same mean distance from the native hosts as where the *T*. *arvense* patches shown in Fig 1E, 1G and 1I were located.

Once the spatial patterns were altered, we analyzed the correlation between the six map attributes to avoid multicollinearity. We then constructed linear models to determine the attributes that explain *R* and *G* using a t-test via the “glm” function in R (ver. 3.0.1, [17]).

We compared the relative speed of the changes of population size and the allele frequency by comparing *R* and *G* for each simulation scenario. If *G* was smaller than *R*, the deleterious allele should be excluded from the population before the butterfly went extinct.

### Sensitivity analysis

We conducted sensitivity analyses to identify the parameters that were fixed in the model, but whose value may have a disproportionate impact on the output. The parameters tested included adult lifespan, immigration, oviposition probability on a host, survival until adulthood, initial population size, and initial allele frequency. We compared the observed relative change in population growth rate resulting from a ±3% change in each parameter. For adult lifespan, we changed the mean of the probability distribution. A large difference from the simulation with observed parameter values indicated that the model had a high sensitivity to a change in a particular parameter. Since our simulation includes stochasticity, population growth rate comparisons are the means of 50 simulation replicates. We started the sensitivity simulations with 50 individuals except for those testing the effects of initial population size, which started with ±3% of 50 individuals. We ran the model for 30 generations to calculate the population growth rate. The sensitivity of allele frequency was analyzed in the same manner.

## Results

### Spatial pattern of *T*. *arvense*

We first ran the model with the observed plant distribution. Butterflies visited both native hosts and *T*. *arvense*. Female population size generally decreased. The average growth rate *R* to generation 100 was 0.989 ± 0.005 SD. The growth rate of the simulation run with identical parameters but lacking *T*. *arvense* (simulation #1 in Fig 3 and Fig 3) was 1.000 ± 0.003 SD. This difference in *R* suggested that the population decrease in the simulation with the observed distribution was due to the mortality of eggs on *T*. *arvense*. The Z_accept_ allele was not excluded from the population over 100 generations (average rate of allele frequency change *G* = 0.997 ± 0.005; see also Fig 3). This result agrees with the ongoing oviposition on *T*. *arvense* in the field [9]. Note that our simulation included immigration, which also occurs in the field.

Because there was a high correlation between *patchnum* and *area* (Spearman's ρ = −0.91, p < 0.0001), we removed *patchnum* from the linear model. The ANOVA of the linear model [*R* = *cover* + *dist* + *area* + *thdist* + *shape*] showed significant effects of all attributes (Table 2A). Next, we conducted an ANOVA to see if there were any relationships between the rate of allele frequency change *G* and the population growth rate *R*. Thus we analyzed the model [*G* = *R*] and found a significantly positive effect of *R* (Table 2B).

**Table 2:** Results of the t-test of the linear models.

Variable	Coefficient (estimate ± SD)	t	p
(a) *R* = *cover* + *dist* + *area* + *thdist* + *shape*			
*cover*	−0.23 ± 6×10^−3^	−37.703	<10^−15^
*dist*	1.00×10^−4^ ± 4×10^−6^	22.772	<10^−15^
*area*	3.55×10^−6^ ± 5×10^−7^	7.871	<10^−14^
*thdist*	−6.92×10^−5^ ± 9×10^−6^	−7.403	<10^−12^
*shape*	−3.04×10^−3^ ± 3×10^−4^	−8.871	<10^−15^
(b) *G* = *R*			
*R*	0.61 ± 0.047	13.204	<10^−15^

**Fig 4:**
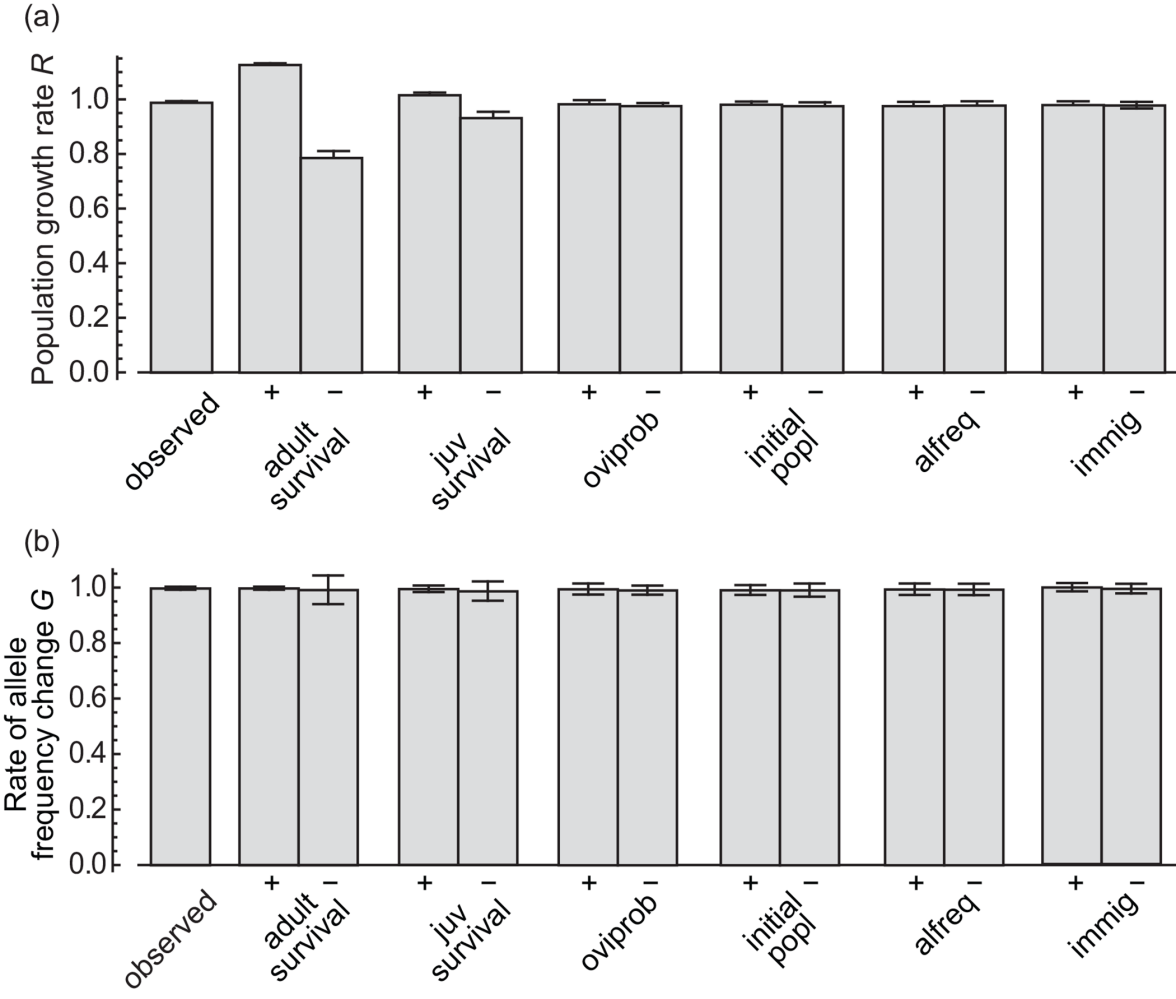
Change of butterfly population growth rate with 4 spatial attributes of *T*. *arvense* distribution that significantly affected butterfly population dynamics: “cover” (a), “dist” (b), “thdist” (c) and “shape” (d). Symbols represent different levels of “cover”, i.e., the proportion of habitat occupied by *T*. *arvense* to the total habitat occupied by the host plants; closed circle: <3%, open circle: <5%, triangle: <10%, diamond: <20%.

The population growth *R* generally decreased with increased proportion of *T*. *arvense*, i.e, “*cover*” (Table 2, Fig 4A). The same trend was observed with increasing “*thdist*”, i.e., the distance between *T*. *arvense* patches (Table 2, Fig 4C). Conversely, increasing the distance from a *T*. *arvense* patch to the nearest native host (“*dist*”) raised the population growth rate (Table 2, Fig 4B). The population growth rate showed sigmoidal increase with increased “*dist*”. All the population growth rates became larger than 0.997 when “*dist*” was increased to 147 m or longer. This sharp change of population growth rate was observed particularly when the proportion of *T*. *arvense* (“*cover*”) was 4 or 8 times higher than observed (shown by triangles and diamonds in Fig 4B, respectively; also see S1 Fig). For example, when “*cover*” was 8 times higher than observed, increasing the mean distance between *T*. *arvense* and the nearest native host by 10.2 m (from 136.5 to 146.7 m) without changing other spatial attributes caused a drastic increase of the population growth rate from 0.968 to 0.981 (simulations #37 and #35 in Table 2 and S1 Table, also see Fig 4B and S1 Fig). The population of 150 becomes extinct at generation 155 under *R* = 0.968 (because 150 × 0.968^155^ < 1) and at generation 262 under *R* = 0.981. The population growth rate showed a U-shaped response to the shape index (Fig 4D), although that could be due to the small number of simulations at intermediate values. Simulations with relatively compact patches (small shape index) generally had higher population growth rates.

The mean *R* was larger than the mean rate of allele frequency change *G* in 1 simulation out of 40, and none of the mean *R*s exceeded *G* + SD (Fig 3). Therefore the rate of population growth was always smaller or almost equal to the rate of allele frequency change. The mean *G* ± SD of all the simulations overlapped with the mean *G* ± SD of the “observed” simulation (Fig 3). The simulations were started with *p* = 0.5 in both sexes, which makes the Hardy-Weinberg equilibrium of allele frequency in the population = (2*p*_*male*_^2^ + 2*p*_*male*_ (1−*p*_*male*_) + *p*_*female*_) / 3 = 0.5. Thus, in all simulations Z_accept_ frequency, *p* did not depart from equilibrium.

### Sensitivity analysis

**Fig 5:**
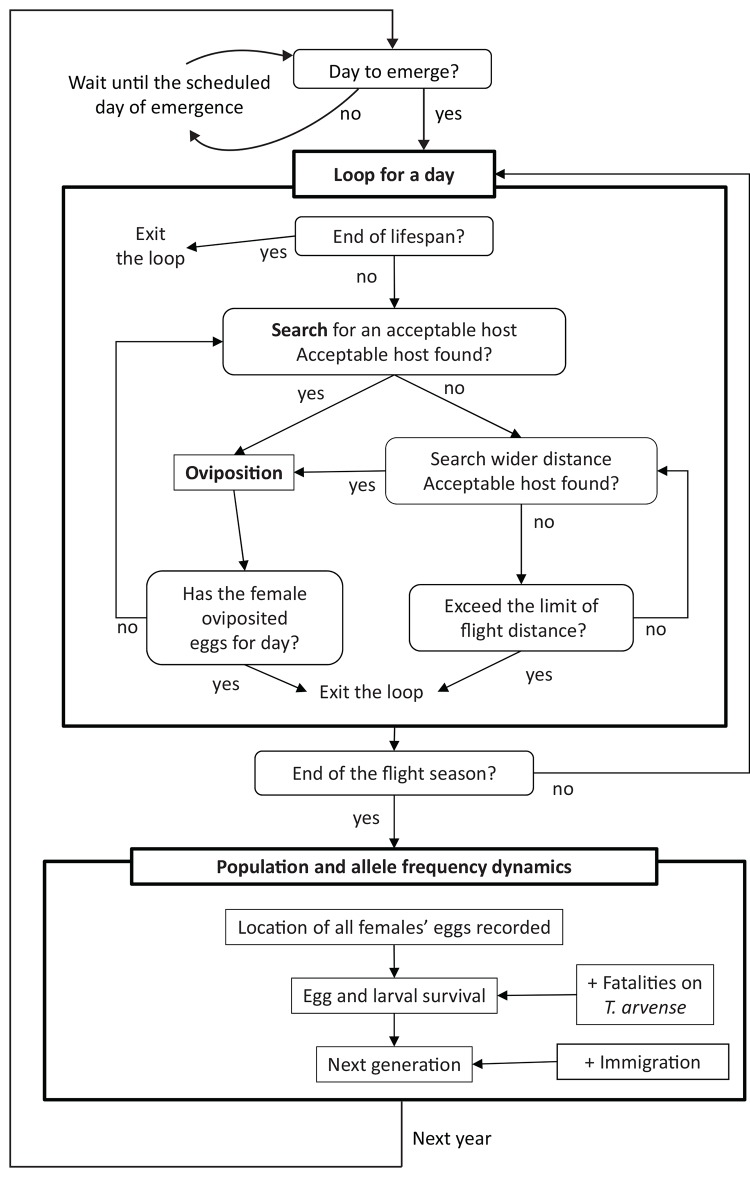
Results of the sensitivity analysis. Bars show the (a) mean population growth rate and (b) mean rate of allele frequency change of simulations with each of the following parameters increased or decreased by 3% (indicated by “+” and “−”, respectively) from the observed value shown in Table 1; “observed”: no parameters were changed; “immig”: immigration rate; “oviprob”: oviposition probability; “alfreq”: initial allele frequency; “juvsurvival”: survival until adult; “lifespan”: lifespan of adult females; “initial popl”: initial population size. The parameters are shown in the order of largest to smallest difference between the means of “+” and “−”except for “observed”. Error bars show SD.

The population growth rate and the rate of allele frequency change showed the highest sensitivities to adult lifespan (Fig 5). Survival until adulthood had the second largest impact on the population growth rate. The remaining simulation parameters did not greatly alter population growth or allele frequency.

## Discussion

The population growth rates of the native butterfly, *R*, obtained from our simulations with different spatial distributions of the detrimental, non-native plant generally decreased with increases in the presence of the non-native, as we expected. The results generally agreed with Ruckelshaus et al. [11]’s IBM done at a coarser-grained scale, which showed that the probability of an ideal organism reaching a new suitable habitat was greatly affected by the amount of suitable habitat and slightly affected by habitat size or shape. In their simulations, although the effect was relatively small, the longer the habitats were, the higher the probability of reaching a new habitat. Reaching a new, suitable habitat in Ruckelshaus et al [11]’s IBM likely results in an increase of population size, whereas reaching a new *T*. *arvense* patch in our IBM reduces the butterfly’s population size. Therefore smaller population growth rates with more elongated patches in our IBM are consistent with Ruckelshaus et al. [11]’s result. They, as well as Fahrig [10], also found that the probability of reaching a new habitat increased more with many smaller habitats than a few larger habitats. In our case, reaching the non-native’s patches more often should reduce the population growth rate. Our simulations showed that the sizes of the non-native host plant patch on the native butterfly’s population dynamics had a positive effect, therefore the number of non-native patches should have a negative effect (due to a strong negative correlation between the size and the number of the non-native’s patch), which was consistent with the results of previous studies. Smaller patches also increased the probability of *P*. *rapae*, a congeneric of *P*. *macdunnoughii*, leaving a patch [18]. The butterflies in our IBM were able to leave a non-native’s patch and visit another one more often when there were smaller, and therefore a larger number, of non-native patches.

Fahrig and Paloheimo [18] also showed that increasing the distance between patches decreases the probability of finding new patches by female *P*. *rapae*. In our case, there was a sharp increase of population growth rate with increasing the distance between the detrimental, non-native plant and the native host plants. Low probability of finding new patches of native host plants might have promoted the quarantine and exclusion of the allele for accepting the non-native host by keeping the female butterflies with the allele inside the non-native patches, while the females without the allele occupied the native host patches.

This result also highlights the importance of considering resource distribution and animal movement at the same, appropriate scale. We found a sharp change in the population growth rate over a 10 m difference in the distance between native and non-native host patches, with a threshold around 147 m. This change became obvious when the proportion of *T*. *arvense* patches to the total host patches was 4 times or more than observed (i.e., >10%), but was not as clear when the percentage of *T*. *arvense* was 1.5 times higher than observed (i.e., 4.5%). Since the total area occupied by the host plants (including *T*. *arvense*) observed in 2000 was 1.3 ha [9], 4.5% of that corresponds to 0.06 ha, whereas 10% corresponds to 0.13 ha. Thus a difference in the total area of *T*. *arvense* as small as 0.07 ha (i.e., 700 m^2^) made the effect of “*dist*” distinct or not. These thresholds cannot be detected using a general, coarser-grained assessment. This “functional grain” [19] scale may also depend on the species’ interactions with the habitat heterogeneity [20]. In our case of a butterfly with a relatively small home range and a patchy host plant well mixed with other plants, a detailed investigation on the host plant distribution was necessary to assess the effect of the invasive host. However, if another invasive species that makes large, dense patches like *C*. *cordifolia* (see Fig 1A) comes into the system, a coarser-grained census of the plant distribution may be adequate to match the size of the patches and the spatial resolution [21].

The population growth rate, *R*, had a positive effect on the rate of the *p* allele frequency change, *G*. That is, when the population shrank, the frequency of Z_accept_ declined. In addition, all the simulations showed *R* almost equal to or smaller than *G* (Fig 3). The population went extinct in some simulations even after the Z_accept_ allele was excluded, probably because the population was already too small to recover when Z_accept_ disappeared. Thus, in the race between extinction and loss of the detrimental allele, extinction won. This resembles the case of race between adaptation and extinction suggested by Maynard Smith [22], which plays an important role in “evolutionary rescue” [23]. Evolutionary rescue refers to a situation where a decreasing population recovers and escapes from extinction due to an increase of adaptive allele frequency. If the population decreases too rapidly, it may cross below a threshold population size where it is susceptible to extinction due to demographic stochasticity, and adaptation cannot rescue it. To examine when the population can be rescued by evolution, Gomulkiewicz and Holt [24] and Orr and Unckless [25] both analyzed mathematical models of a population which experienced a sudden and severe environmental change, and whose fitness was affected by a phenotype characterized by one locus, which was analogous to our simulation. They concluded that the probability of evolutionary rescue was affected by the initial population size and the initial level of maladaptation. In our case, the sensitivity analysis showed no remarkable effect of the initial population size nor of the initial level of maladaptation (i.e., initial frequency of *p*) on the population growth and allele frequency change (Fig 5). This result indicates that the initial population size may not affect the fate of population, or that the initial population might be too small to be rescued. Theoretical models of closed systems suggested a threshold population size which can be rescued as 10–100 individuals [24]. Similarly, the initial frequency of *p* may have no effect on the fate of butterfly, or *p* = 0.5 could be too high to be excluded. We should note, however, that our model described the simplest evolutionary system, whereas discussions concerning evolutionary rescue, such as the theoretical models cited above, include mutations, environmental fluctuations and other factors that may change the speed of evolution in our case as well.

Immigration can also play an important role in maintaining Z_accept_. Immigration could rescue the population either through an increase in population size (demographic rescue) or through an increase in the frequency of the adaptive allele (genetic rescue) [23]. However, field data on our system suggested that gene flow between the focal population and neighbors may be high, such that the immigrant source and focal populations may have similar allele frequencies ([15], Boggs personal observation), as assumed in our simulation that resulted in population decrease. Even when we ran simulations assuming that all immigrants were from a population in which the Z_accept_ frequency was 0, the population growth rate increased from that of the observed simulation scenario but Z_accept_ allele was not excluded within 150 generations (results not shown). Thus, the population is more likely to be rescued demographically rather than genetically by immigrants.

Interestingly, extinction won the race with adaptation in our simulations even though the locus for acceptance of the detrimental host plant was on the sex chromosome, which has been shown to evolve more rapidly than loci on autosomes when the allele in question is lethal [26]. The complicated inheritance system of oviposition preference on the sex chromosome must have contributed to slow the evolution in reality: the Z_accept_ allele is lethal not for the individual who carries Z_accept_ but only for those of his/her offspring who were born on the non-native host plant. The other, lucky offspring who were born on the native plant grow, reproduce and pass Z_accept_ to future generations. This modification of the timing of lethality may well slow elimination of the Z_accept_ allele from the population sufficiently to also result in decreases in population size, which then lead to an eco-evolutionary spiral to extinction.

Indeed, these results were obtained with the simple assumption of 1-locus-2-allele system, although a mathematical model suggests that similar results obtain for multiple loci system [24]. We also lack information on survival from egg to adulthood except for larval survival. In our IBM, this survival was set to maintain the butterfly population at a stable size when the non-native plant was absent. If survival is higher in reality, the population can grow faster than in our IBM and the maladaptive allele may be excluded. More details of the genetics, as well as demography, are required to assess the speed of evolution in the field and gain additional insight into this eco-evolutionary feedback. In addition, there is another possibility for evolutionary rescue in real life: the butterfly could become able to utilize *T*. *arvense* as a host plant. A population of the congeneric, *P*. *oleracea*, may have evolved to utilize a detrimental invasive plant at least within 60–100 generations [27, 28]. Our simulation did not consider evolution of larval utilization ability.

We used an IBM, which is stochastic, even though animal movements are often approximated by deterministic mathematical models such as time-dependent partial differential equations (PDEs). We also developed a PDE that assumed the simple diffusion of butterfly density to compare butterfly movement with our IBM, and obtained almost identical results (see S2 Appendix for details). However, the results of the IBM were obtained faster than the numerical solution of the PDE on a fine-grained scale. In addition, the IBM was able to incorporate phenology, oviposition preference, and genetics in an intuitive manner, unlike a mathematically complicated PDE. An IBM in general is a powerful tool that allows direct assessment of the population-level impacts of variation among individuals [29, 30]. An IBM can include complex rules both of movement determined by encounters with resources that are heterogeneously distributed (e.g., dispersal step size, frequency and direction) [31, 32]. In fact, incorporation of spatial information into IBMs (Spatially Explicit Population Models, SEPMs; [33] has yielded numerous insights into spatial ecology ([30] and references therein). In spite of the benefits, to our knowledge this study is the first example of SEPMs which studied eco-evolutionary dynamics affected by biological invasions.

An IBM that simulates a realistic, non-ideal, idiosyncratic system can also be a powerful tool for species conservation and landscape management [34]. Our study provided specific thresholds for abundance and distribution of an invasive species that may cause severe population declines. Given that *T*. *arvense* occupies disturbed areas, our simulations can help with the development of a habitat management plan to preserve *P*. *macdunnoughii*. For example, given that *shape* had a significantly negative effect on population growth, it might be advantageous to reclaim or develop habitat in a compact shape rather than in an elongated shape when other attributes such as total area are the same.

Overall, our results demonstrate the importance of analyzing habitat structure at the scale experienced by an organism to understand population dynamics. The habitat structure includes the distribution of resource, phenology and amount of immigration. This is true not only for understanding the effects of invasive species, but also for effects of, e.g., climate change [35]. Studies such as ours that integrate behavior, habitat use and individual and population responses to realistic niche structures will give insights both into the fate of specific populations and into the concepts that govern those fates.

## Supporting Information

S1 Appendix Parameter estimation. (DOCX)

S2 Appendix Individual based model and partial differential equation. (DOCX)

S1 Fig Mean population growth rate obtained from the IBM, and proportion of *T*. *arvense* in the habitat (“*cover*”) and mean distance from a *T*. *arvense* patch to its nearest native host patch (“*dist*”) of the plant distribution used in that simulation. (DOCX)

S1 Table Spatial patterns of *T*. *arvense* simulated by the IBM. (XLSX)
