# Correction: Fine-Grained Distribution of a Non-Native Resource Can Alter the Population Dynamics of a Native Consumer

**DOI:** 10.1371/journal.pone.0145874

**Published:** 2015-12-21

**Authors:** Mifuyu Nakajima, Carol L. Boggs

Figs 2, 3, 4 and 5 are incorrect. The figures are out of order and associated with the wrong legend. The authors have provided corrected versions here.

**Fig 2:**
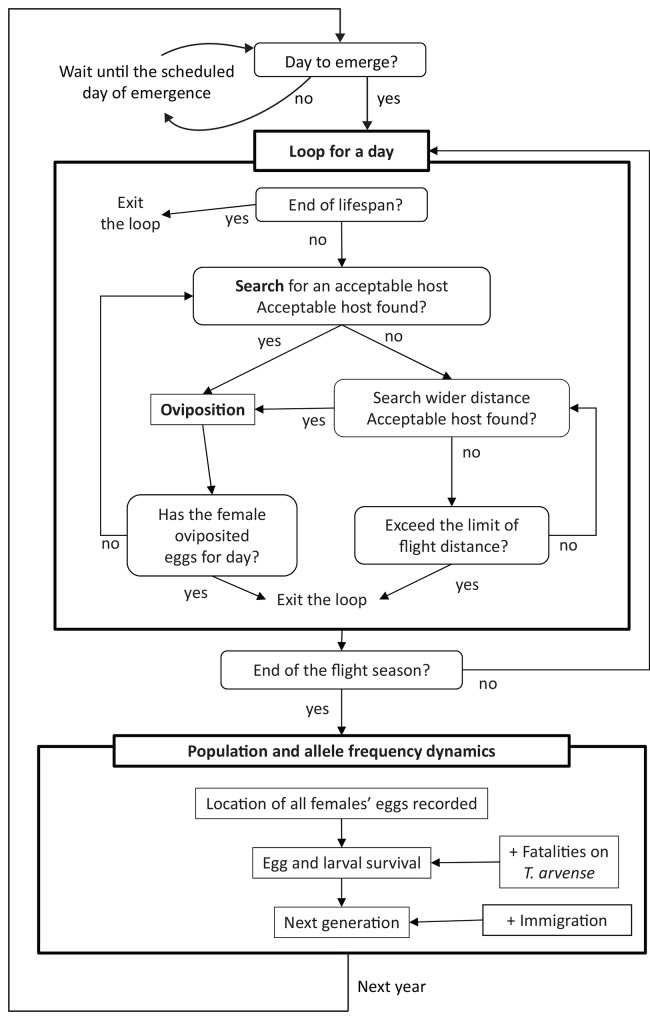
Simulation flow chart for the IBM.

**Fig 3:**
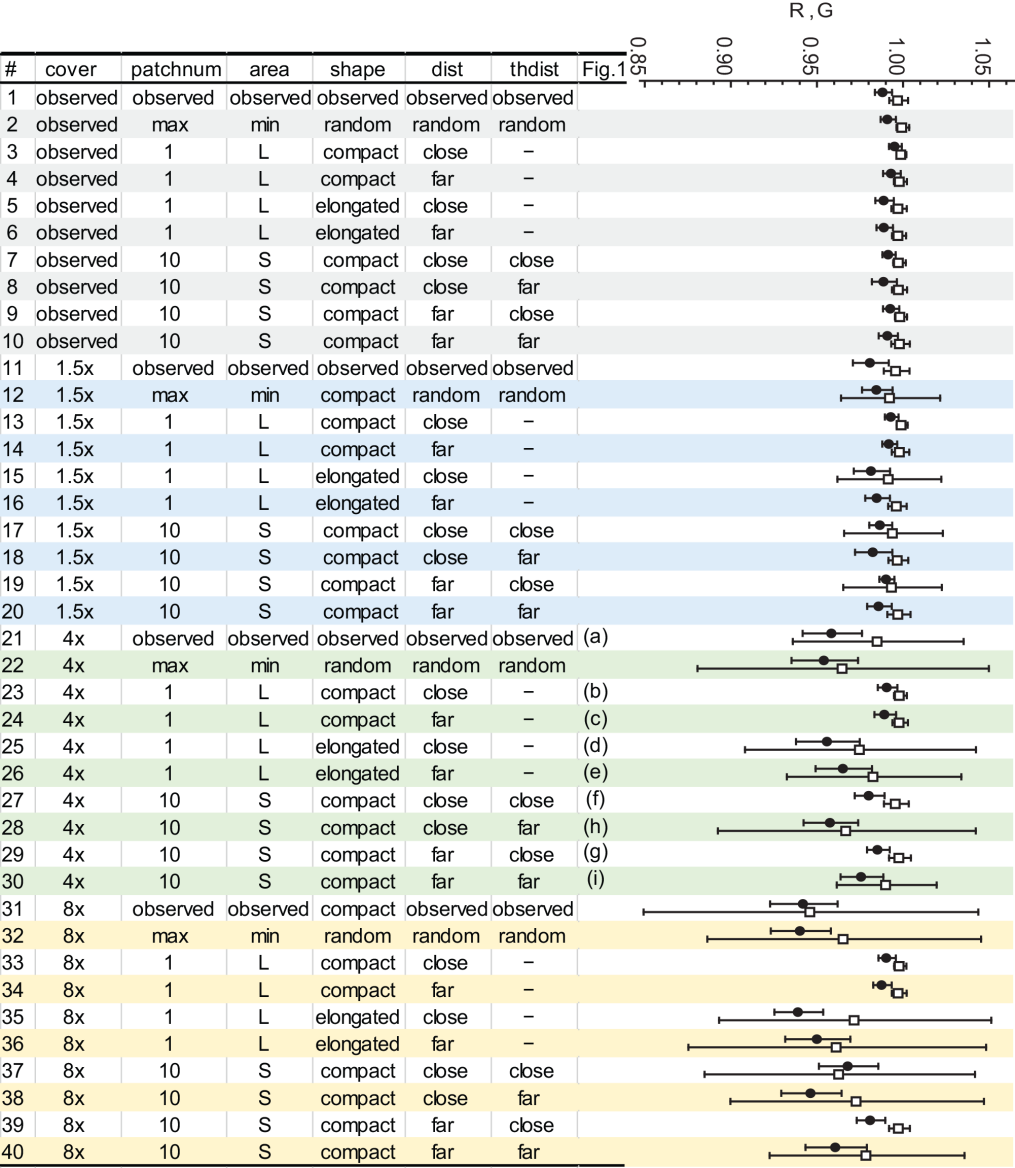
Spatial patterns of *Thlaspi arvense* simulated by the IBM (left) and the population growth rate *R* and the rate of allele frequency change *G* of each simulation, shown in closed circle and open square, respectively (right). The right-end column of the table shows the corresponding panel in Fig 1.

**Fig 4:**
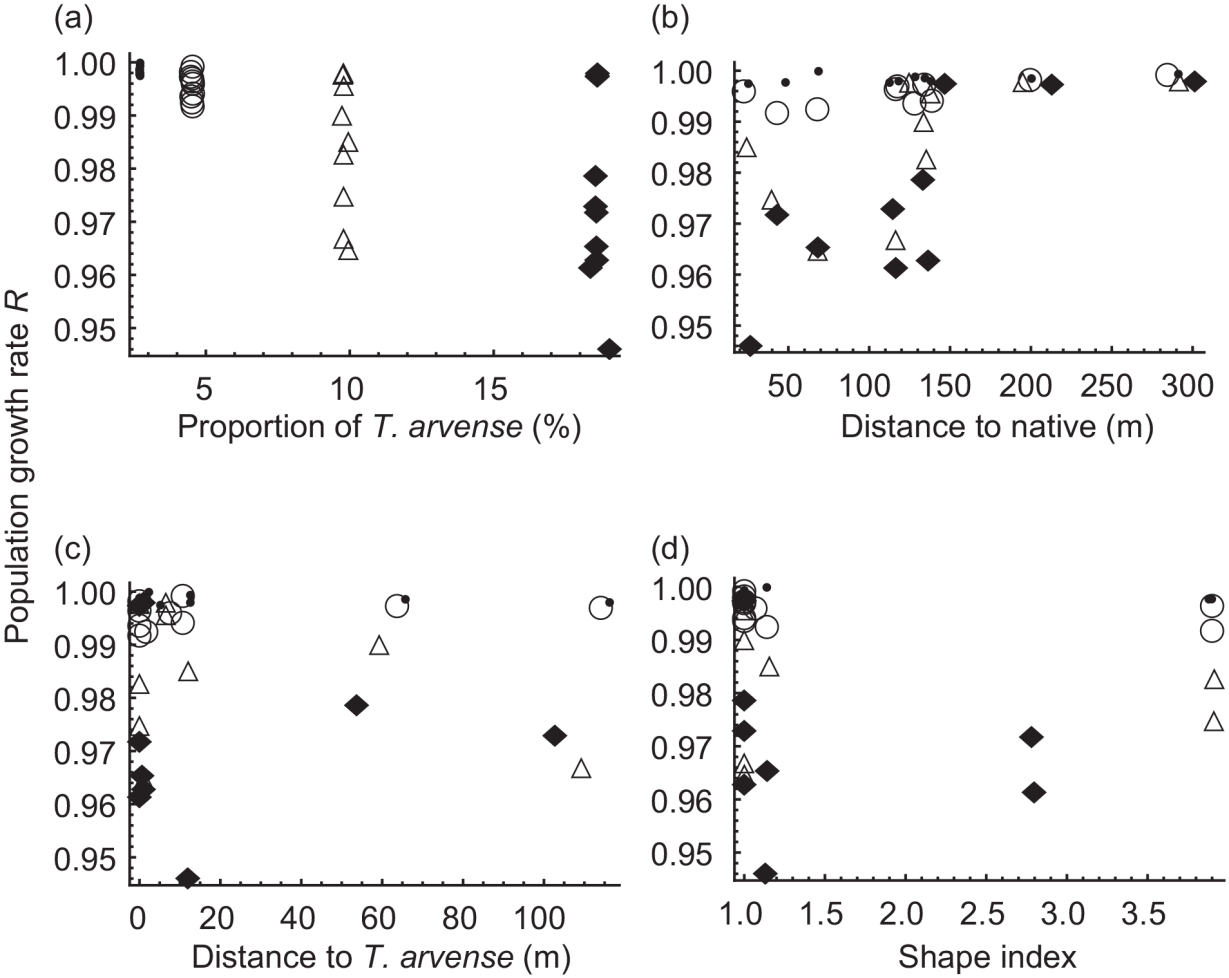
Change of butterfly population growth rate with 4 spatial attributes of *T*. *arvense* distribution that significantly affected butterfly population dynamics: “cover” (a), “dist” (b), “thdist” (c) and “shape” (d). Symbols represent different levels of “cover”, i.e., the proportion of habitat occupied by *T*. *arvense* to the total habitat occupied by the host plants; closed circle: <3%, open circle: <5%, triangle: <10%, diamond: <20%.

**Fig 5:**
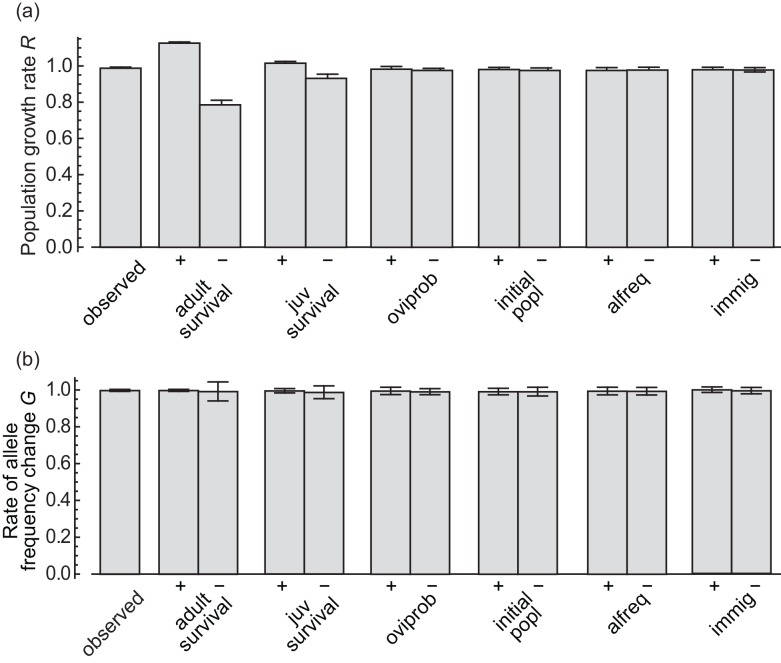
Results of the sensitivity analysis. Bars show the (a) mean population growth rate and (b) mean rate of allele frequency change of simulations with each of the following parameters increased or decreased by 3% (indicated by “+” and “−”, respectively) from the observed value shown in Table 1; “observed”: no parameters were changed; “immig”: immigration rate; “oviprob”: oviposition probability; “alfreq”: initial allele frequency; “juvsurvival”: survival until adult; “lifespan”: lifespan of adult females; “initial popl”: initial population size. The parameters are shown in the order of largest to smallest difference between the means of “+” and “−”except for “observed”. Error bars show SD.
