# Supplementary material for: Fine-Grained Distribution of a Non-Native Resource Can Alter the Population Dynamics of a Native Consumer
Source: PLoS One. 2015 Nov 17;10(11):e0143052. doi: 10.1371/journal.pone.0143052 (PMC4648569; doi:10.1371/journal.pone.0143052)
Supplement: S1 Appendix — (DOCX) [file pone.0143052.s001.docx]

Fine-grained distribution of a non-native resource can alter

the population dynamics of a native consumer

Mifuyu Nakajima, Carol L. Boggs

# Supporting Information

## S1 Appendix: parameter estimation

### Female emergence

Percentage of the total new adult females eclosing on each day was obtained from mark-release-recapture data collected in 1971 and analyzed by Nakajima et al. [[1](#_ENREF_1)]. The females with a wing wear score of 2.0 were regarded as newly emerged individuals (see [1] for the definition of wing wear score). This data set contained 234 females including 59 individuals with a score of 2.0. We calculated the proportion of score 2.0 individuals to total females caught for each day and fitted a β-function (Fig. A in S1 Appendix, Table 1 in text). In the simulation, the proportion of females that eclosed each day relative to the total females that survived from eggs laid by the previous generation until eclosion was determined by this β-function.

Figure A. Percentage of the total new adult females eclosing each day. We fitted the proportion of new adults to the β-function (estimated α =1.75, β =8.28).

### Flight distance

Previous observations of female *P. macdunnoughii* in the field showed that the turning angles between flights were random and that the average flight distances in wet, intermediate, and dry habitats were 1.8, 2.3, and 3.7 m, respectively [[1](#_ENREF_1)]. As the length of one cell was 0.25 m, we assumed that females would stop within 8, 10, and 15 cells in wet, intermediate, and dry habitats, respectively, of the current location. From Kareiva and Shigesada’s [[2](#_ENREF_2)] report on the flight distances of *P. rapae* during sequential oviposition, we estimated the average distance as 1.53 m. We therefore assumed that females that were on acceptable hosts, had eggs and were motivated to oviposit moved within 6 cells in our simulation.

To our knowledge, there are no data giving total flight distance per day for Pieris butterflies, i.e., the summed distance of all short movements. Therefore we used fine-scale observations of butterfly movements. A previous study [[1](#_ENREF_1)] showed that the average flight speed between landings was 0.29 m/s (s.d. = 0.56), with a minimum and maximum of 0.001 and 2 m/s, respectively. Assuming that females fly nearly continuously and that the average flight time per day at the elevation of the RMBL is 4 h (~10:00 h to 14:00 h; see also [[3](#_ENREF_3)]), the minimum and maximum distances per day were 16.38 and 28,000 m, respectively. Therefore, we derived a daily flight distance of 4134.54 m (s.d. = 8022.98) randomly from a normal distribution with the minimum and maximum limits. The estimated minimum limit (16.38 m) thus was at 30.4 percentile, whereas the estimated maximum limit (28,000 m) was at 99.9 percentile. The probability distribution of the daily flight distance was thus skewed to short distances as shown in Figure B in S1 Appendix.


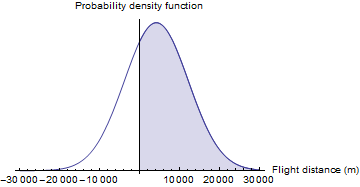


Figure B. Probability distribution of daily flight distance. The daily flight distance was randomly selected from the shaded area between the minimum (16.38 m) and the maximum (28,000 m) distances of a normal distribution with mean = 4134.54 m and s.d. = 8022.98.

### Daily egg load

The change in daily egg load with the day since mating in Swedish *Pieris napi* followed a log-normal curve [[4](#_ENREF_4)]. Therefore we first fitted a log-normal distribution to the daily egg load of monandrous females of Swedish *Pieris napi* as a function of the time since first mating recorded by Wiklund et al. [[4](#_ENREF_4)] and obtained a mean ± s.d. of 1.69 ± 0.66 (ANOVA, F = 83.11, df = 3, 20, p < 10^−10^). Then we adjusted the distribution to a maximum daily egg load of 90, which was recorded for the focal *P. macdunnoughii* population [[5](#_ENREF_5)] by multiplying the distribution by 483.7. We should note that in our simulation a female mated immediately after eclosion, and started ovipositing from the day of eclosion if the butterfly encountered an acceptable host plant and was motivated to oviposit.

### Lifespan

Adult female survival rates of *P. macdunnoughii* at RMBL were estimated using mark-release-recapture over a 5 km transect in 1971, 2000, and 2010 [[1](#_ENREF_1)]. The average of the estimated daily survival rates was 0.87. This number included both death and emigration. We assumed a normal distribution of daily survival rates in the female population, and that the maximum daily survival observed (0.94) deviated from the average by 2×s.d. of the distribution. To determine the lifespan for a female butterfly, we first obtained a random number from this normal distribution of daily survival rates, and then calculated the lifespan as a rounded integer of –ln(daily survival rate) ^–1^ days (see [[1](#_ENREF_1)] for calculation). The average lifespan was calculated as –(ln0.86) ^–1^ = 6.63, therefore 7 days. If the lifespan exceeded the flight season, the butterfly was set to die at the end of the last day of the flight season.

Survival from egg to adulthood included egg hatchability, larval survival, and pupation and adult emergence success. Survival from egg to pre-pupa on native hosts was 1.6% averaged over 2 years and two host species [[6](#_ENREF_6)], whereas pupation and emergence success are unknown. Therefore, we estimated total survival until adulthood from the simulation as the value that maintains the butterfly population size with the absence of *T. arvense* for 100 generations. From 50 simulation runs, we obtained a survival probability of 0.00651 yielding a population growth rate *R* = 1.00000 ± 0.00263SD.

## References

1. Nakajima M, Boggs CL, Chew FS, Cummings A, Bowsher JH. Dynamics and structure of a native Pieris population in the presence of a non-native, toxic larval host plant. J Lep Soc. 2014; 68: 175-184.

2. Kareiva PM, Shigesada N. Analyzing insect movement as a correlated random walk. Oecologia. 1983; 56: 234-238.

3. Kingsolver JG. Thermoregulation and flight in *Colias* butterflies: elevational patterns and mechanistic limitations. Ecology. 1983; 64: 534-545.

4. Wiklund C, Kaitala A, Lindfors V, Abenius J. Polyandry and its effect on female reproduction in the green-veined white butterfly (*Pieris napi* L.). Behav Ecol Sociobiol. 1993; 33: 25-33.

5. Ehrlich AH, Ehrlich PR. Reproductive strategies in the butterflies: I. Mating frequency, plugging, and egg number. J Kansas Entomol Soc. 1978; 51: 666-697.

6. Nakajima M, Boggs CL, Bailey S, Reithel J, Paape T. Fitness costs of butterfly oviposition on a lethal non-native plant in a mixed native and non-native plant community. Oecologia. 2013; 182: 823-832.
