# Supplementary material for: Fine-Grained Distribution of a Non-Native Resource Can Alter the Population Dynamics of a Native Consumer
Source: PLoS One. 2015 Nov 17;10(11):e0143052. doi: 10.1371/journal.pone.0143052 (PMC4648569; doi:10.1371/journal.pone.0143052)
Supplement: S2 Appendix — (DOCX) [file pone.0143052.s002.docx]

Fine-grained distribution of a non-native resource can alter

the population dynamics of a native consumer

Mifuyu Nakajima, Carol L. Boggs

# Supporting Information

## Appendix S2: individual based model and partial differential equation

Partial differential equations (PDEs) ignore some components that individual-based models (IBMs) can incorporate, such as individual variation (e.g., oviposition preference, genotype, age, and experience), as well as temporal variation (e.g., phenology of host plants and female eclosion). Therefore, to compare the movements of female butterflies in the two models, we modified the IBM assumptions from the basic model described in the text as follows. First, the diffusion of Z_reject_ and Z_accept_ females were separately simulated. Second, we assumed that all plants are available throughout the butterfly flight season. In addition, the PDE should not be run continuously for multiple generations because the offspring genotype of a simulated female is determined solely by her mate. At the beginning of each generation, the number of Z_reject_ and Z_accept_ female offspring and their initial locations should be determined by combining the results of the PDE for Z_reject_ and Z_accept_ of the previous generation. In the PDE, *v_i_*(*x*,*y*,*t*), the probability density for type *i* (*i* = Z_reject_ or Z_accept_), the female butterfly's location at coordinate (*x*,*y*) in a 2-dimentional space at time *t*, is described as:

*∂v_i_* (*x*,*y*,*t*)/ *∂t* = *d_i_*(*x*,*y*) (*∂*^2^*v_i_* (*x*,*y*,*t*)/ *∂x*^2^ + *∂*^2^*v_i_* (*x*,*y*,*t*)/ *∂y*^2^) Eq. S1

where *d_i_*(*x*,*y*) denotes the diffusion coefficient for female type *i* at each coordinate (*x*,*y*). The value *d_i_* depends on aridity and host type, consistent with the movement rule in the IBM (8, 15, 10, and 6 cells/s for wet, dry, intermediate, and acceptable host cells, respectively). Note that for cells occupied by *T. arvense*, *d_Zaccept_* = 6 but *d_Zreject_* = 8, 15, or 10. To simplify the numerical analysis of the PDE, we reduced the habitat from the one used for the IBM basic model, i.e., the plant distribution observed in 2000, to 62.5 × 62.5 m (250 × 250 cells; Fig. C in S2 Appendix ). We applied a Dirichlet condition *v_i_* (*x*,*y*,*t*) = 0 at all boundaries, which is equivalent to assuming free emigration and no return into the habitat. Different boundary types, such as reflecting Neumann conditions, could also be applied and compared to the IBM, with differences corresponding to the boundary conditions. As a proxy for the number of eggs oviposited on hosts, we accumulated the probability density at host-occupied cells from *t* = 1 until the simulation ended. We obtained the numerical solution of Eq. S1 separately for Z_reject_ and Z_accept_ females using an alternating-direction implicit method (ADI) ([[1](#_ENREF_1)]) and summed the accumulated probability densities on hosts obtained for Z_reject_ and Z_accept_. For the probability distribution at *t* = 0, we randomly selected 50 cells each for Z_reject_ and Z_accept_ from cells occupied by native hosts, and set *v_i_*(*x*,*y*,0) = 1 for those cells. The simulation was stopped after 1 generation (average lifespan, 7 days) or when the total probability density across the habitat became less than 1. We also changed our IBM from the basic model as follows: all individuals were born on day 1, all individuals lived an average lifespan (7 days), all individuals carried the average number of eggs, all individuals with eggs oviposited on all acceptable hosts they encountered, all the hosts were available throughout the year, all individuals flew the average flight distance/day every day, individuals emigrated when the next destination was outside the habitat, and no individuals returned to the habitat.

Figure C. Habitat used in the simulations. Orange: *T. arvense*, green: native hosts, light gray: dry, black: wet, gray: intermediate aridity. Numbers indicate the cell number.

We ran the IBM starting with 100 individuals (50 Z_reject_s and 50 Z_accept_s) for one generation 500 times. As a proxy for female movement, we recorded the number and spatial distribution of oviposited eggs. We used the average number of eggs oviposited in each cell at each *t* for repeated simulations. The same habitat as well as the initial locations of the female butterflies were used for both the PDE and IBM. The simulation was aborted when all the individuals emigrated.

The probability density of females in the PDE was distributed heterogeneously, corresponding to the heterogeneous diffusion coefficient (Fig. D in S2 Appendix). The movement patterns observed in the PDE and IBM were consistent. The final egg densities in each cell in the PDE and the IBM were significantly correlated (F_1,62498_= 528702, p < 10^−29^, r^2^ = 0.89). The ratio of eggs oviposited on *T. arvense* by Z_accept_ females to the total eggs oviposited was 0.050 in the PDE and 0.047 in the IBM. Thus, the butterfly movement pattern in our IBM was well matched with the diffusion process. Note that we obtained the same results when we analyzed 50 randomly selected IBM simulations (from 500 simulations), the same number as used in our analyses shown in the main text.


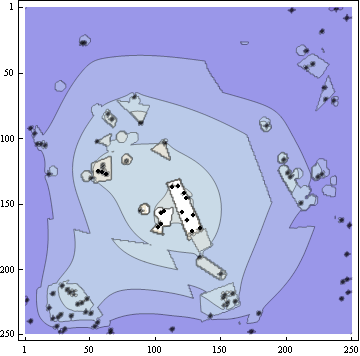


Figure D. Contour plot of the accumulated population density of Z_0_ + Z_1_ at the end of simulation obtained by PDE at *t* = 1121. Lighter color indicates higher density; black dots indicate the initial distribution.

The number of iterations, which is often crucial for the model run time, differed between PDE and IBM. In the PDE, which can be solved only numerically, habitat was discretized and the equation was solved for each cell. Therefore the number of cells (or “mesh size”) influenced the run time. In the IBM, the number of individuals in the simulation affected the run time. Because we were interested in the effect of habitat heterogeneity on a fine-grained scale, the number of cells was large. On the other hand, the number of iterations in our IBM was smaller than the number of individuals. Thus, we selected all of the individuals for the same habitat condition (host, wet, dry, or intermediate) from the entire habitat and added a random distance (different numbers among individuals) to the current location, then updated the new locations for all individuals. Thus we treated the coordinates of the selected individuals as a large matrix and calculated the subsequent destinations while reducing the number of iterations of the IBM. In our case, therefore, the IBM simulations were complete before those of the PDE when both models were run on the same computer with the scenarios described above.

## References

1. Press WH, Teukolsky SA, Vetterling WT, Flannery BP. Numerical recipes in C. 1992. Cambridge: Cambridge University Press.
