# Supplementary material for: Fine-Grained Distribution of a Non-Native Resource Can Alter the Population Dynamics of a Native Consumer
Source: PLoS One. 2015 Nov 17;10(11):e0143052. doi: 10.1371/journal.pone.0143052 (PMC4648569; doi:10.1371/journal.pone.0143052)
Supplement: S1 Fig — (DOCX) [file pone.0143052.s003.docx]

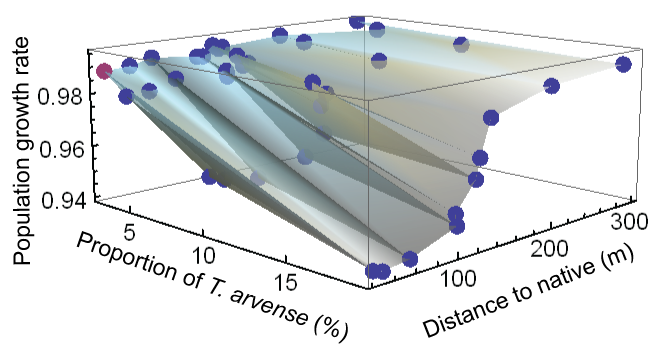


S1 Fig. Mean population growth rate obtained from the IBM, and proportion of *T. arvense* in the habitat (“*cover*”) and mean distance from a *T. arvense* patch to its nearest native host patch (“*dist*”) of the plant distribution used in that simulation. Each dot represents each simulation, where red dot shows the “observed” simulation (#1 in Fig. 3) and the blue dots show others. Change of the population growth rate with the distance to native became more abrupt when the proportion of *T. arvense* was higher.
